# Elaphuri Davidiani Cornu Improves Depressive-Like Behavior in Mice and Increases Neurotrophic Factor Expression in Mouse Primary Astrocytes via cAMP and ERK-Dependent Pathways

**DOI:** 10.3389/fphar.2020.593993

**Published:** 2020-11-16

**Authors:** Yue Zhu, Mengqiu Liu, Suchen Qu, Cheng Cao, Chongqi Wei, Xue-er Meng, Qianyin Lou, Dawei Qian, Jin-ao Duan, Yuhua Ding, Zhengxiang Han, Ming Zhao

**Affiliations:** ^1^ Jiangsu Key Laboratory for High Technology Research of TCM Formulae and Jiangsu Collaborative Innovation Center of Chinese Medicinal Resources Industrialization, Nanjing University of Chinese Medicine, Nan Jing, China; ^2^ Jiangsu Province Dafeng Milu National Nature Reserve, Dafeng, China; ^3^ Department of Neurology and Rehabilitation, Shanghai Seventh People’s Hospital, Shanghai University of TCM, Shanghai, China

**Keywords:** elaphuri davidiani cornu, depression, neurotrophic factor, astrocytes, animal medicine

## Abstract

Elaphuri Davidiani Cornu (EDC) is the natural shedding horn of *Elaphurus davidiauus* Millne-Edwards that was used by people in ancient China for maintaining physical and mental health. We evaluated the antidepressant effect of EDC using depression-like animal models and explored possible mechanisms in mouse primary astrocyte cultures. We found that aqueous extracts of EDC significantly improved depression-like behavior in a mouse model of depression. The extracts enhanced expression of nerve growth factor and brain-derived neurotrophic factor neurotrophic factors in mouse prefrontal cortex and hippocampus tissues. In the mouse primary astrocyte cultures, the EDC aqueous extracts significantly increased the neurotrophic factor expression both at the transcriptional and protein levels. EDC extracts might exhibit these functions by regulating matrix metalloprotein-9 of the nerve growth factor and brain-derived neurotrophic factor metabolic pathways and might enhance expression of neurotrophic factors via the cAMP- and ERK-dependent pathways. We confirmed this possibility by showing the effects of related inhibitors, providing scientific evidence that supports the utility of EDC in the development of drugs to treat major depressive disorders.

## Introduction

There are 6,008 Chinese medicinal materials recorded in *Zhong Yao Da Ci Dian* (Great Dictionary of Chinese Medicine) (Zhao et al., 2006). Among them, Elaphuri Davidiani Cornu (EDC) is definitely a special one with a unique fate. It is the natural shedding horn of the Père David’s deer, also known as elaphure or Milu (*Elaphurus davidiauus* Millne-Edwards). Elaphure is not only the endemic species in China but also a precious medicinal animal. The horn, fat, and meat of elaphure can all be used as medicinal materials; their application history stretches over at least a 1000 years. EDC is the most frequently used medicinal part of the elaphure and was first described by Tao Hong-jing in *Ming Yi Bie Lu* in 450 A.D. The first formula of EDC was recorded in *Beiji Qianjin Yaofang* by “Medical King” Sun Si-miao in 652 A.D. (Li, 1997). According to the historical records, the function of EDC has been summarized as “warming the kidney and strengthening *yang,* nourishing *ying* and supplementing the essence*,* strengthening bones and muscles, and activating blood circulation” (Song, 2002). However, as elaphure became extinct in China in the 1900s, the medicinal application of EDC ceased. Up to 1986, “the World Wide Fund for Nature (WWF)” donated 39 elaphures (13 males, 26 females) to the Chinese government that been kept in the Jiangsu Dafeng National Nature Reserve to restore elaphure wild populations in their native habitat. Through more than 30 years of efforts by the Chinese government and experts, the elaphure population has increased by nearly 70-fold and now consists of over 6,600 animals. Four wild-elaphure populations and other sporadic groups have formed. The restoration of the elaphure population has made it possible to restore the medical application of EDC.

EDC was used in ancient China to treat various diseases including hypo-immunity, osteoporosis, rheumatoid arthritis, and others. Various studies have been carried out *in vivo* or *in vitro* to validate the EDC function or reveal the mechanism of action. EDC has been reported to induce cellular and humoral immunity and macrophage function. It was also found to induce the transformation of bone marrow cells and the proliferation of hematopoietic progenitor cells in a cyclophosphamide-induced blood deficiency mouse model and increase the bone density, mineral content, and the AKP (alkaline phosphatase) level in the ovariectomized rat model. EDC also exerted an anti-aging effect on d-galactose-induced senescence in mice by increasing the SOD (superoxide dismutase) activity and decreasing the content of MDA (malondialdehyde) and monoamine oxidase in the liver and brain. These modern studies confirmed that EDC could indeed be a valuable medical material.

Based on the ancient medical literature, EDC could also be used to improve depressive mood, however, this function has never been studied and validated (Li, 1997). Therefore, we evaluated the effect of EDC on improving depression mood using in animal and cell models and explored the active substances involved and their mechanisms of action. Here we report the effect of water and ethanol EDC extracts on improving depressive mood using the tail-suspension and forced-swimming mouse models, and on mouse astrocyte primary culture *in vitro* to evaluate the regulation of neurotrophic factors. We separated the EDC extract by ultrafiltration into molecular-weight fractions and treated astrocytes to explore the presence of active substances. Our findings may indicate possible mechanisms of EDC activity in improving depressive mood and offer a scientific basis for EDC drug development.

## Materials and Methods

### Preparation of Extracts

The natural shedding of EDC was collected from Jiangsu Dafeng Milu National Nature Preserves. The morphology of EDC and the voucher number can be found in Supplementary Fig.1 Water and ethanol extracts of EDC were prepared as follows. Pulverized EDC (15 g) was mixed with distilled water (500 ml) and refluxed for 2 h. The supernatant was removed and the residue was added to 500 ml distilled water for second reflux. The supernatants were combined and concentrated by lyophilization to produce solid material. The ethanol extract was prepared as above but using 85% ethanol. The extracts were characterized using HPLC-MS/MS as previously reported (Li et al., 2013). The representative chromatogram could be found in Supplementary Fig.2. The composition of the extracts is shown in Supplementary Table S1. This composition sets up the chemical standards of EDC extracts for further pharmacological and biological studies.

### Animals and Housing Conditions

Male ICR mice (7–8 weeks old, 18–22 g) were purchased from Qinglong Mountain experimental Animal Culture Co. Ltd. ICR mice were raised in SPF surroundings in the animal center of Nanjing University of Chinese Medicine. All procedures for treating animals were following the Guide for the Care and Use of Laboratory Animals approved by the Institutional Animal Care and Use Committee. The experimental procedures also conformed to the guidelines of the “Principles of Laboratory Animal Care” (NIH publication No. 80-23, revised 1996).

### Drug Treatment

The mice were divided into six treatment groups and contained eight individuals randomly assigned to each group. The control group animals were given saline intragastrically. The positive control group was given fluoxetine (4 mg/kg) (Zhu et al., 2017). The four test groups were given aqueous or ethanol extracts of EDC at dosages (expressed as the crude material) of 2 g/kg/day and 6 g/kg/day, respectively. Animals were treated for 7 days.

For determination of related signaling pathway on astrocyte cultures, PKI (protein kinase A inhibitor, 10 nM) and U0126 (ERK inhibitor, 2 μM) were treated on astrocytes for 3 h before exposures to EDC extract, forskolin (10 μM), and TPA (100 nM) (Cao et al., 2018).

### Behavioral Tests

The depression-like behavior was evaluated using the tail-suspension tests (TST) and forced-swimming tests (FST). The tail-suspension test was carried out first, with the forced-swimming test performed 24 h later. The experimental details can be found in our previous publication (Cao et al., 2018). In details, individual mouse was suspended in an acoustically and visually isolated chamber. Animal activities were captured by a video camera. The total time of immobility during the last 4 min in a 6 min testing periods was analyzed by software. FST was carried out after 1 day of TST. Similarly, individual mouse was moved in an acoustically and visually isolated chamber and placed in a clear glass tank (40 cm high and 20 cm in diameter) filled with 30 cm of water (22–23°C) and allowed to swim for 6 min. The activities of tested mouse were also recorded by a video camera. The time of mouse floating in the water without struggling were calculated as immobile time and total immobile time during the last 4 min of the 6 min testing period were analyzed by ANY-maze software (Stoeling Co.Ltd., United States).

### Astrocyte Primary Culture

Astrocyte primary culture was prepared from the cortex tissue of postnatal ICR mice at day 1. Cell-culture reagents for astrocytes were purchased from ThermoFisher Scientific (Waltham, MA). The cortex tissues were isolated and trypsinised. The cells were centrifuged and the supernatants were removed. The pellets were suspended and cultured. The cultures were further purified by shaking after 90% of confluence was reached. The cultures were shaken at the speed of 200 rpm/min for 8 h to remove oligodendrocytes and microglia as reported in our previous publication (Zhu et al., 2013).

### ELISA Assays

The expression of nerve growth factor (NGF) and brain-derived neurotrophic factor (BDNF) were determined by ELISA assays. The tissues of the prefrontal cortex and the hippocampus were removed from the sacrificed mice and homogenized. After centrifugation, the supernatant was separated and analyzed by mouse NGF ELISA and mouse BDNF ELISA kits (Aviscera Bioscience, Santa Clara, CA). The protein content was expressed in ng/g wet-tissue weight as reported in our earlier publication (Zhu et al., 2017).

### Western Blot Analysis

The kinase phosphorylation of signaling pathways was analyzed as follows. The total protein content was extracted from the astrocyte primary culture, separated by 8% polyacrylamide gels, and semi-quantified by SDS (sodium dodecyl sulfate) PAGE (polyacrylamide gel electrophoresis) as reported previously (Zhu et al., 2016a). Primary antibodies used were: rabbit polyclonal anti-Erk (extracellular signal-regulated kinase) (4,695, 1:2,000; Cell Signaling Technology), rabbit polyclonal anti-pErk (4,370, 1:2,000; Cell Signaling Technology), rabbit polyclonal anti-CREB (cAMP response element binding protein) (1:2,000; Cell Signaling Technology), rabbit polyclonal anti-pCREB (1:2,000; Cell Signaling Technology), and rabbit polyclonal anti-GAPDH (glyceraldehyde-3-phosphate dehydrogenase) antibody (1:2,000; Cell Signaling Technology). The secondary antibody used was anti-rabbit IgG antibody conjugated with horseradish peroxidase (1:5,000, Cell Signaling Technology). Immunoreactivity was visualized by ECL (electrochemiluminescence) reagent (Tianneng Co. Ltd., Shanghai, China), and blots were visualized and compared on the imaging system (ChemiDoc™ XRS+, Bio-Rad, Hercules, CA).

### Real-Time Quantitative PCR Analysis

Transcriptional levels of kinases accounting for the synthesis and degradation of neurotrophic factors were determined by quantitative PCR analysis. The experimental details can be found in our earlier publications (Zhu et al., 2016b). Firstly, the total RNA from mice astrocyte cultures was extracted by Trizol reagent (Invitrogen, Carlsbad, CA). The concentrations and purity of RNA were determined by DS-11 Spectrophotometer (DeNovix, Wilmington, DE) and the quality of RNA was evaluated by the ratio of absorbance at 260–280 nm and ranged from 1.9 to 2.1. Total RNA samples were reverse-transcribed using the cDNA obtained using EasyScript One-Step gDNA Removal and cDNA Synthesis SuperMix kit (Transgen Biotech, Beijing, China). Real-time quantitative PCR analysis was carried out by using TransStart Top Green qPCR SuperMix kit (Transgen Biotech, Beijing, China) on Applied Biosystems 7,500 fast real-time PCR system (Thermal Fisher Inc., Foster City, CA). Transcript levels of target genes were quantified by using the ΔΔCt value method. The primers of genes are listed in Supplementary Table S2.

### Data Analysis

The comparison of multiple groups was performed by using one-way or two-way ANOVA followed by a Bonferroni post hoc analysis if appropriate (version 13.0, SPSS, IBM Corp., Armonk, NY). Normal-distribution test of data was carried out before the ANOVA. All data are expressed as the mean ± SEM, where *n* = 8. Differences were statistically labeled as significant [*] for *p* < 0.05 or highly significant [**] for *p* < 0.01.

## Results

### Extracts of Elaphuri Davidiani Cornu Exhibited an Antidepressant Effect on Mice

**FIGURE 1:**
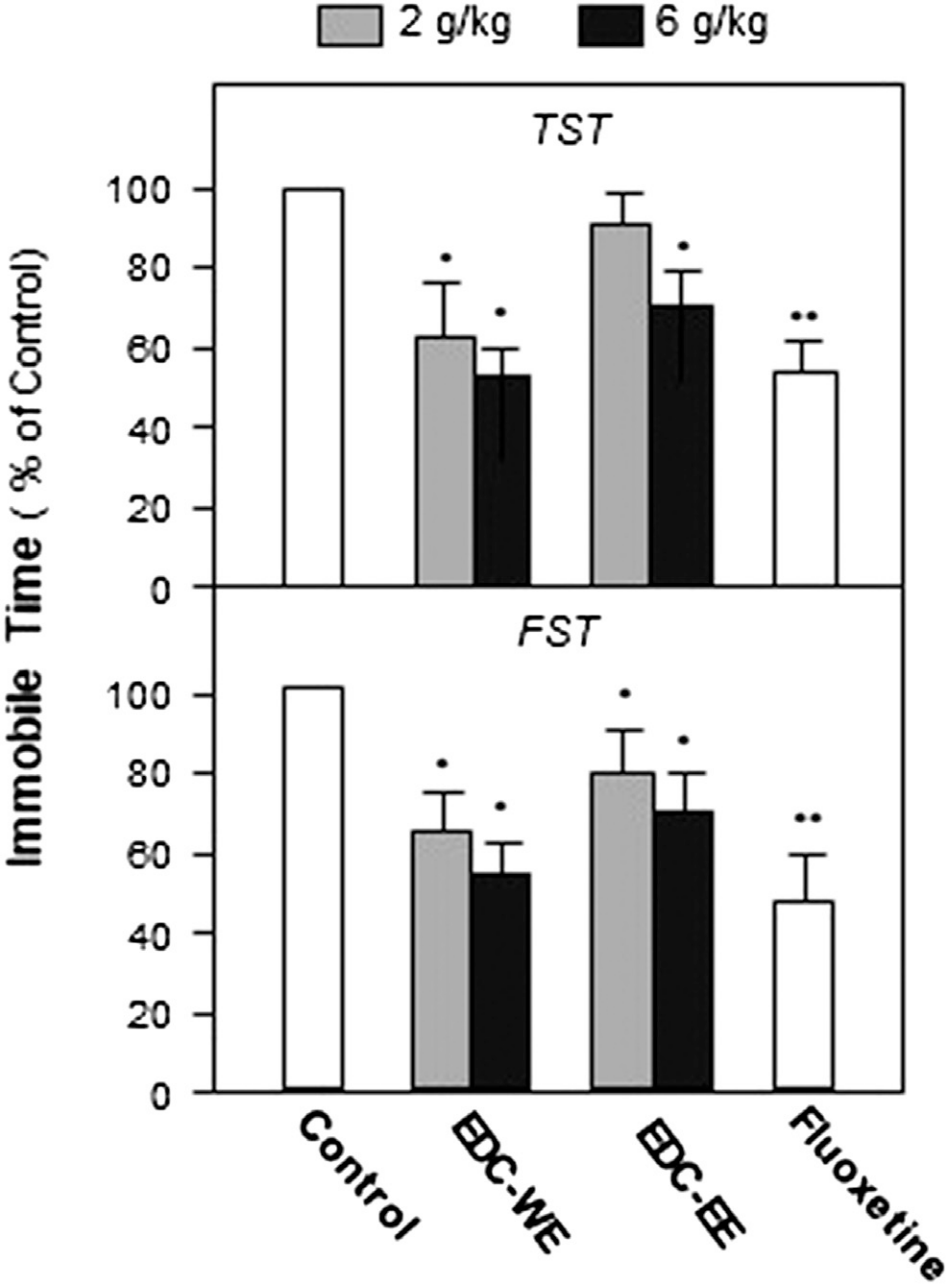
Extracts of Elaphuri Davidiani Cornu (EDC) improved the depression-like behaviors of mice. After 7-days treatment with the EDC aqueous (EDC-WE) and ethanol extracts (EDC-EE), groups of mice were subjected to the tail-suspension (TST) and forced-swimming tests (FST). Fluoxetine was used as a positive control. Each EDC extract was administered at two doses of 2 and 6 g/kg of the converted to crude EDC material). Values are expressed in percentages of the control group (no-drug treatment group), as mean ± SEM (*n* = 8). *p < 0.05, **p < 0.01 (compared with the control group).

To evaluate the antidepressant effect and explore the active components of EDC, the aqueous and 70% ethanol extracts were prepared and intragastrically administered to the depression-model animals. TST and FST, two widely used behavioral tests for antidepressant screening, were used to measure the depression-related responses after treatment with EDC extracts for seven consecutive days. As displayed in Figure 1, both the aqueous and 70%-ethanol extracts of EDC decreased the immobile time of mice in the FST compared with the control group (*p* < 0.05). The aqueous extract of EDC at two dosages both decreased the immobile time of TST significantly compared with the control group while the ethanol extracts exhibited a positive effect only at the high dose. In the FST, the aqueous and ethanol extracts of EDC at two dosages all significantly decreased the immobile time compared with the control group mice. Therefore, the aqueous extract of EDC exhibited a higher antidepressant effect than the 70% ethanol extract.

Extracts of EDC increased the expression of the neurotrophic factors in the prefrontal cortex and hippocampus tissues of mice.

**FIGURE 2:**
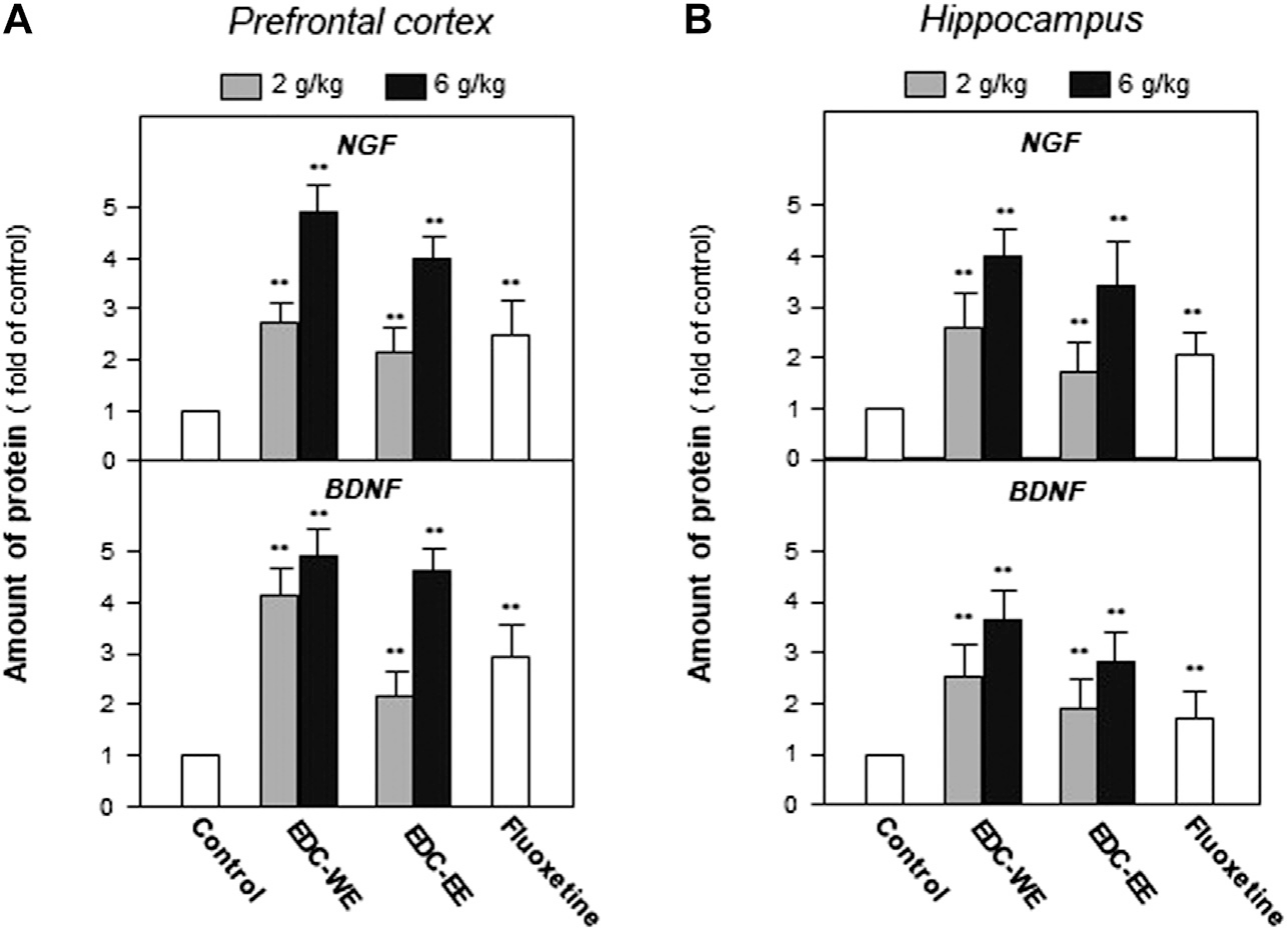
Extracts of Elaphuri Davidiani Cornu (EDC) increased the expressions of neurotrophic factors in the prefrontal cortex and hippocampus tissues of mice. **(A)**: Mice were given aqueous (EDC-WE) and ethanol extracts (EDC-EE) of EDC by intragastric administration at the stated doses—for 7 days. The amounts of nerve growth factor and brain-derived neurotrophic factor were determined in the tissues of the prefrontal cortex of the sacrificed mice. Fluoxetine was used as a positive control (4 mg/kg/day). **(B)**: The samples were the same as in **(A)** and the amounts of nerve growth factor and brain-derived neurotrophic factor were determined in the tissues of the hippocampus of the sacrificed mice. Values are expressed as the percentage of the control group (no drug treatment group), as mean ± SEM (*n* = 8). *p < 0.05, **p < 0.01 (compared with the control group).

After the behavioral tests were completed, the expression of NGF and BDNF was determined in the prefrontal cortex and hippocampus of sacrificed mice using ELISA. Treatment with a higher dose of aqueous or 70%-ethanol EDC extracts significantly increased the expressions of NGF and BDNF in the prefrontal cortex and hippocampus compared with the no-drug-treatment control group (*p* < 0.01) (Figures 2A). This increase was consistent with the results of the behavioral tests. The aqueous extract of EDC at the higher dose increased up to five-fold the expressions of both NGF and BDNF at a level superior to fluxeotine. The effects of EDC extracts at higher dosages were all higher than at the lower dosage. The aqueous extract exhibited a better effect than the ethanol extract. Similar effects were found in the hippocampus tissue (Figures 2B). Taken together, EDC significantly increased the expressions of NGF and BDNF in the prefrontal cortex and hippocampus of mice.

Extracts of EDC increased the expressions of neurotrophic factors in mouse astrocytes.

The mechanism of the EDC effect to increase the expressions of NGF and BDNF was examined further using the astrocyte primary-culture cell model. Though NGF and BDNF can be secreted from neurons and glial cells in brain, EDC aqueous exracts are mainly water-soluble peptides and cannot cross blood brain barrier and affect neurons directly. More realistically, the peptides might be absorbed into blood and act on astrocytes that make up the blood brain barrier. Therefore, we selected astrocytes as the cell model and treated EDC aqueous extract on astrocyte cultures. The EDC aqueous extract was selected for two reasons. Firstly, compared with the EDC ethanol extract, the aqueous extract showed a stronger effect on promoting the expression of NGF and BDNF in mice. Secondly, the aqueous extract is the major clinical application form of EDC in traditional Chinese medicine.

**FIGURE 3:**
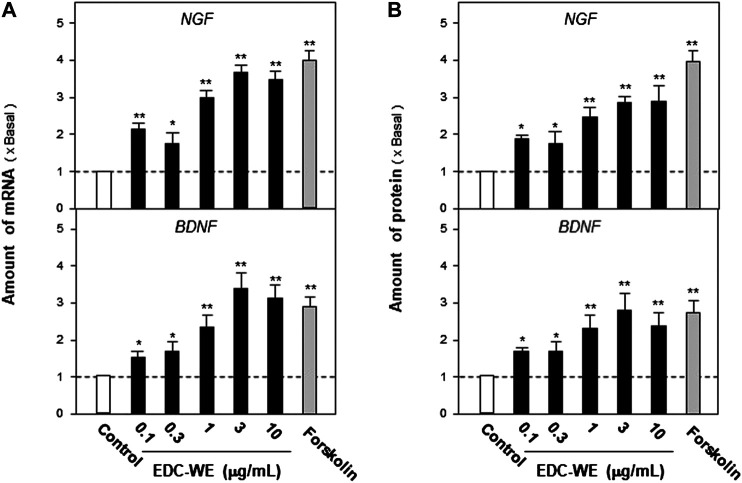
The aqueous extracts of Elaphuri Davidiani Cornu (EDC) increased the neurotrophic factor expression in mouse astrocyte primary cultures. **(A)**: The primary mouse astrocyte cultures were treated with the aqueous extract (EDC-WE) at 0.1–10 μg/ml concentrations for 24 h. The mRNA expression levels of nerve growth factor and brain-derived neurotrophic factor were determined by qPCR analysis. **(B)**: The drug treatment with EDC-WE was the same as in **(A)** and the expression of nerve growth factor and brain-derived neurotrophic factor was determined by ELISA. Forskolin (10 μM) was used to treat the positive-control group. Data are expressed as a fold-change relative to the positive control group (no-drug treatment) as mean ± SEM, where *n* = 5. *p < 0.05, **p < 0.01 (compared with no-treatment control group).

Astrocyte primary cultures that reached 90% confluence were treated with the ECD aqueous extract at 0.1–10 μg/ml concentration for 48 h and the transcriptional levels of NGF and BDNF were determined. As displayed in Figures 3A, the aqueous extract increased the transcriptional levels of NGF and BDNF by more than three-fold compared with no-drug treatment group (*p* < 0.01). The expression of NGF and BDNF was confirmed by the protein levels using ELISA. As displayed in Figures 3B, the expression of NGF and BDNF proteins increased up to three-fold after treatment with the EDC aqueous extract. The EDC aqueous extract promoted the expressions of NGF and BDNF in a dose-dependent manner.

The aqueous extract of EDC promotes the expression of neurotrophic factors in mouse astrocyte primary culture via the cAMP- and ERK-dependent pathways.

We explored the possible signaling pathways involved in the effect of the EDC aqueous extract in promoting the expressions of NGF and BDNF in the mouse astrocyte culture. It is well known that both the cAMP- and Erk-dependent signaling pathways are involved in the production of NGF and BDNF. We co-treated astrocytes with the EDC aqueous extract and the inhibitors of cAMP- or Erk-dependent signaling pathway to observe possible changes in the NGF and BDNF expressions and evaluated the extent of phosphorylation of the critical kinases on the two pathways.

**FIGURE 4:**
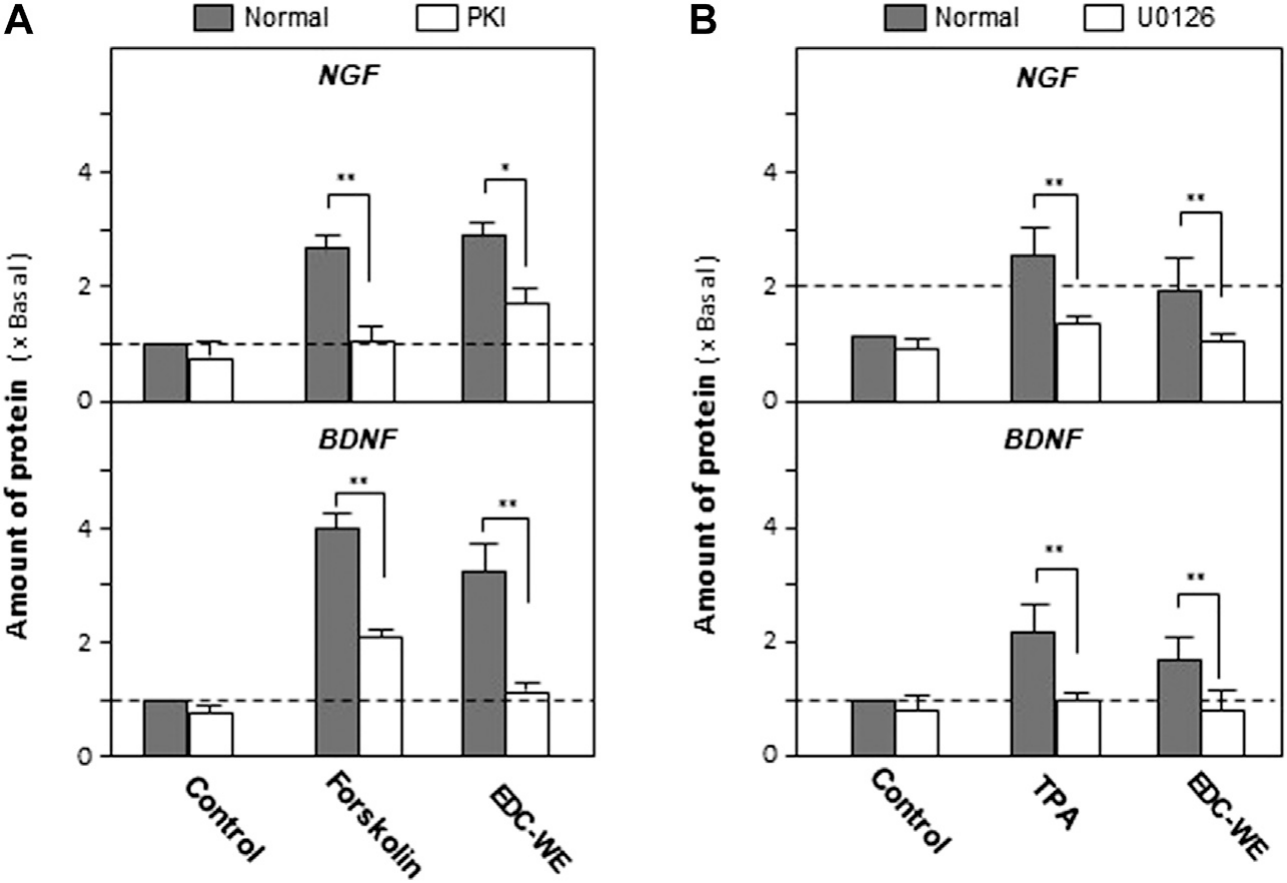
Aqueous extracts of Elaphuri Davidiani Cornu (EDC) increased the expression of nerve growth factor and brain-derived neurotrophic factor in mouse astrocyte primary cultures via the cAMP- and ERK-dependent pathways. **(A)**: The mouse astrocyte primary cultures were pre-treated with PKI (10 nM) for 3 h and then treated with forskolin (10 μM) and EDC-WE (3 μg/ml) for 24 h. The expression of nerve growth factor and brain-derived neurotrophic factor were determined by ELISA. **(B)**: The mouse astrocyte primary cultures were pre-treated with U0126 (2 μM) for 3 h and then treated with TPA (100 nM) and EDC-WE (3 μg/ml) for 24 h. The expression of neurotrophic factors was determined by ELISA. Data are expressed as a fold-change relative to the control group (no-drug treatment) in mean ± SEM, where *n* = 5. *p < 0.05, **p < 0.01.

As displayed in Figures 4A, the treatment with forskolin, an agonist of the cAMP-dependent pathway, significantly increased the expression of NGF and BDNF compared with the no-drug treatment group (*p* < 0.01). PKA (Protein kinase A) inhibitor significantly reversed the increase induced by forskolin. EDC aqueous extract also significantly induced the expressions of NGF and BDNF. Treatment with PKI weakened the increase of NGF and BDNF expressions in astrocytes treated with the aqueous EDC extract. As shown in Figures 4B, application of TPA, or the aqueous EDC extract significantly increased the expressions of NGF and BDNF while the U0126 inhibitor of Erk reversed the increasing trend.

**FIGURE 5:**
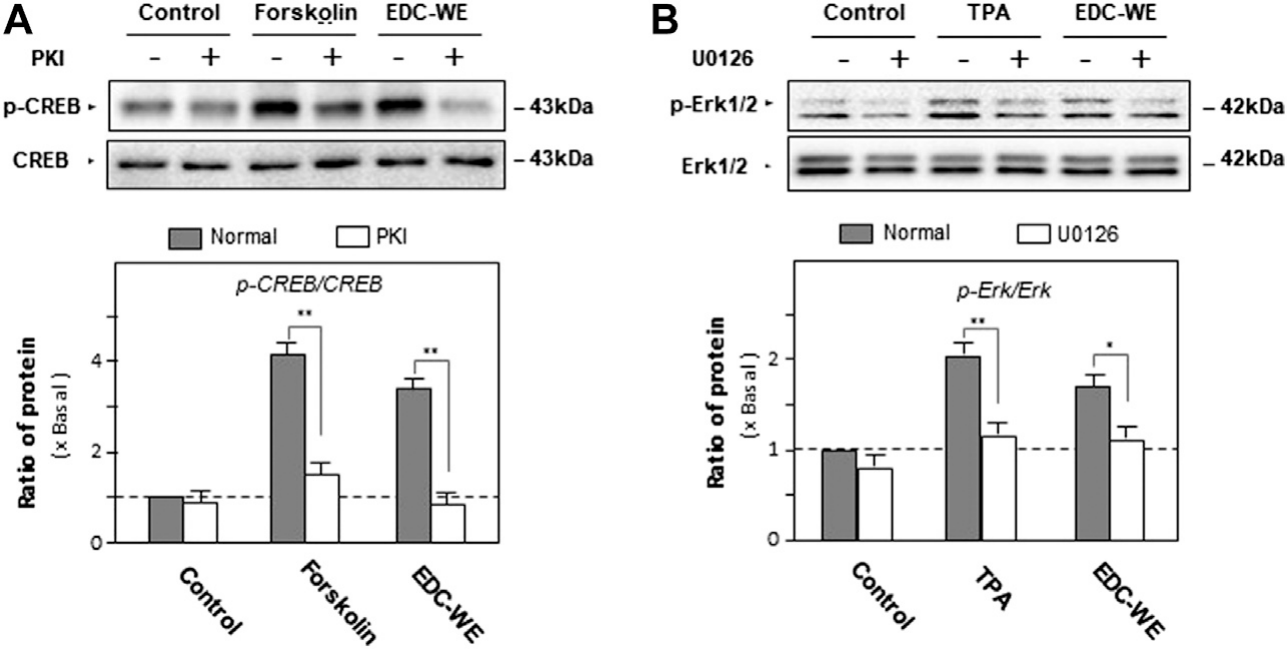
The effect of the aqueous extract of Elaphuri Davidiani Cornu (EDC) on the phosphorylation of kinases of the cAMP- and Erk-dependent pathways in the mouse astrocyte primary cultures. **(A)**: Mouse astrocyte primary cultures were pre-treated with PKI (10 nM) for 3 h and then treated with forskolin (10 μM) and the aqueous extracts (EDC-WE) (3 μg/ml) for 24 h. Protein-expression levels of p-CREB and CREB in astrocyte primary cultures were determined by western blot analysis. Data are expressed as a fold-change relative to the control group (no-drug treatment) in the ratio of p-CREB to CRRB as mean ± SEM, where *n* = 5. **(B)**: Mouse astrocyte primary cultures were pre-treated with U0126 (2 μM) for 3 h and then treated with TPA (100 nM) and the EDC aqueous extract (EDC-WE) (3 μg/ml) for 24 h. Protein-expression levels of p-Erk and Erk in astrocyte primary cultures were determined by western blot analysis. Data are expressed as a fold-change relative to the control group (no-drug treatment) in the ratio of p-Erk to Erk as mean ± SEM, where *n* = 5. *p < 0.05, **p < 0.01.

To validate the roles of the signaling pathways, the phosphorylation of CREB and Erk were examined. As displayed in Figure 5, forskolin and TPA both significantly increased phosphorylations of CREB and Erk. The antagonist PKI and U0126 significantly inhibited phosphorylations of CREB and Erk. The aqueous EDC extract inhibited the phosphorylation of both CREB and Erk. These data suggest that both cAMP- and Erk-dependent pathways play pivotal roles in the increase by the aqueous EDC extract in the expression of NGF and BDNF in mouse astrocytes.

The aqueous extracts of EDC increase the expression of a neurotrophic factor in mouse astrocyte cultures via regulating MMP-9.

**FIGURE 6:**
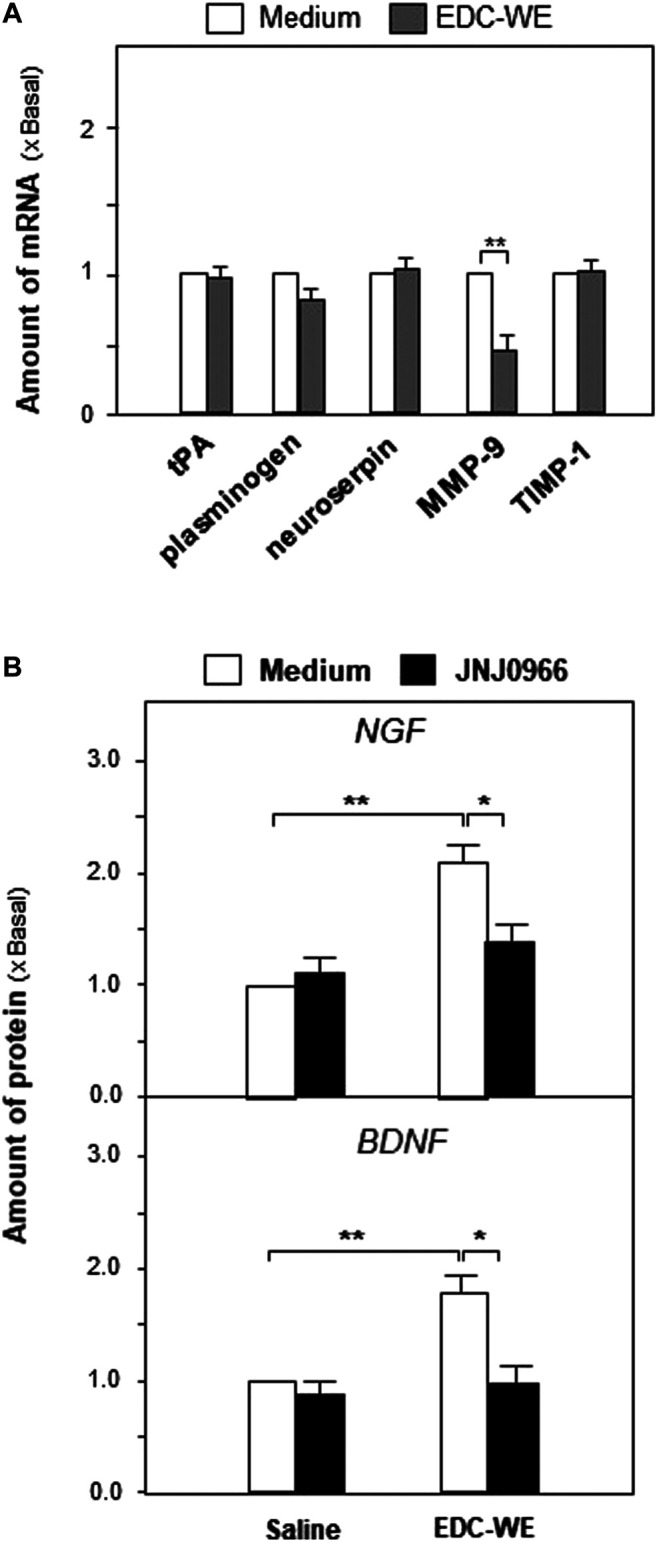
The aqueous extract of Elaphuri Davidiani Cornu (EDC) increased neurotrophic factor expressions via the regulation of the metabolic pathway of neurotrophic factors in mouse astrocyte primary cultures.**(A)**: The transcriptional levels of proteases accounting for neurotrophic factor metabolism were determined by qPCR analysis in mouse astrocyte primary cultures treated with the aqueous extract (EDC-WE) (3 μg/ml) for 24 h **(B)**: Mouse astrocytes were pre-treated with JNJ0966 (MMP-9 inhibitor, 0.4 μM) for 24 h and then treated with the EDC aqueous extract (EDC-WE) (3 μg/ml) for 24 h. The transcriptional levels of neurotrophic factors were determined by qPCR analysis. Data are expressed as a fold-change relative to the control group (no-drug treatment) as mean ± SEM, where *n* = 5. *p < 0.05, **p < 0.01.

We explored further the possible active targets of the EDC aqueous extract in increasing the expressions of NGF and BDNF. The EDC aqueous extract significantly increased the expression of neurotrophic factors in mouse astrocyte cultures after treatment at 3 μg/ml concentration for 24 h. The transcriptional levels of the critical enzymes related to the neurotrophic-factor metabolism were determined. As shown in Figures 6A, the EDC aqueous extract exhibited no observable effect on the expressions of synthesizing enzymes such as tissue plasminogen activator (tPA), plasminogen, and neuroserpin. However, the EDC aqueous extract significantly downregulated the transcriptional levels of matrix metallopeptidase 9 (MMP-9) over two-fold while showing no effect on the level of the tissue inhibitor of metalloproteinase 1 (TIMP-1).

Following up on the results obtained for mRNA levels, we used JNJ0966 (the blocker of MMP-9) to determine whether the EDC aqueous extract regulated the metabolism of neurotrophic factors via MMP-9. As shown in Figures 6B, the increased expressions of NGF and BDNF in mouse astrocytes were significantly downregulated by the treatment with JNJ0966, implying that the EDC aqueous extract might regulate the neurotrophic factor metabolism by modulating MMP-9.

## Discussion

Studies reported here aimed to re-evaluate and excavate the possible scientific values of EDC using modern technologies. Using several depression-like animal models, we found that EDC exerted antidepressant effect via regulating neurotrophic factors such as NGF and BDNF. In particular, the EDC water extract showed a better effect in increasing neurotrophic factor expression compared with the ethanol extract. The data implied that EDC may exhibit neuronal activities.

Neurotrophic factors are important substances in the maintenance of the physiological function of the brain and the pathological process of neurodegenerative diseases and mood disorders. Neurotrophic factors maintain the survival, differentiation, and synaptic formation of neurons. Neurotrophic factors also play an important role in memory formation and emotion maintenance. NGF maintains the survival of cholinergic neurons, and cholinergic neurons are well-known neurons for the formation and maintenance of memory. BDNF enhances neurogenesis, dendritogenesis, and maintains the synapse stability. It also regulates glutamatergic and GABAergic signaling that play an essential role in long-term memory. In Alzheimer’s disease, abnormal regulation and insufficient supply of NGF have been regarded as a crucial pathological process. In major depression disorders, serum BDNF level has been used as a diagnostic parameter and low levels of BDNF were found in the serum of depression patients (Molendijk et al., 2014). Low levels of NGF are also found in the serum of depression patients (Mössner et al., 2007). However, neurotrophic factors are proteins that are easily digested in the gastrointestinal tract and the transcranial administration is not practical in clinically. Therefore, increasing the endocrine supply of neurotrophic factors has become a target for drug development to treat major depressive disorders and Alzheimer’s disease (Iulita and Claudio, 2014).

While the available antidepressants such as fluoxetine and venlafaxine are known to elevate the levels of NGF and BDNF both in the serum of depression patients and in depression-like animal models (Mondal and Fatima, 2019), the long-term use of these antidepressants can cause many side effects. The representative tricyclic antidepressants imipramine can cause anticholinergic adverse reactions such as xerostomia and constipation, central nervous system toxicity such as tremor, dyskinesia and epilepsy, and cardiovascular toxicity such as postural hypotension, tachycardia, conduction block, arrhythmia, and cardiac arrest. Compared with TCAs, serotonin reuptake inhibitors (SSRIs) have relatively fewer side effects and toxicity. However, common adverse reactions of SSRIs include insomnia, nausea, irritability, headache, exercise anxiety, mental tension, and tremor. A long-term medication of SSRIs often leads to loss of appetite or loss of sexual function. More seriously, fluoxetine increases the suicide risk in non-adult depression patients. Therefore, the development of antidepressants from natural medicines and with relatively few side effects has become the focus of attention (Cipriani et al., 2018).

Some natural medicines such as *Crocus sativus*, *Curcuma longa*, *Cuscuta spp.*, *Hypericum perforatum*, *Lavandula spp.*, *Panax ginseng,* and so on (Sarris, 2018) have been shown to have significant antidepressant effects. The types of compounds involved are flavonoid, alkaloid, saponins, monoterpene, and polysaccharide, and others. However, there are very few reports on the antidepressant effects of ingredients from animal medicine (Bahramsoltani et al., 2015). We have found that the powder of EDC significantly improves the depressive behavior in animal models. To study the material basis of the EDC antidepressant activity, we prepared aqueous and alcohol extracts of EDC. We have shown that in animal models, the antidepressant effect of EDC water extract was stronger than that of the alcohol extract. In astrocytes, the main donor of neurotrophic factors in the brain, the enhancing effect of EDC water extract on the expression of neurotrophic factors was significantly higher than that of the alcohol extract. In the current study, we have found that the EDC aqueous extract promotes the expression of neurotrophic factors mainly by inhibiting MMP-9; this differs from the effect of some plant extracts that we have previously found to enhance the expression of neurotrophic factors by promoting the expression of synthase (Zhu et al., 2017). However, MMP-9 plays different role in processing of neurotrophic factors. It degrades mature NGF while converts pro-BDNF to mature BDNF (Mizoguchi et al., 2011). The biological character of pro-neurotrophin is totally different from processed mature neurotrophin. The pro-neurotrophins bind to p75NTR (p75 neurotrophin receptor) to initiate neuronal apoptosis while the mature neutotrophins bind to Trk (tyrosine kinase) receptor to nourish the neurons (Lu et al., 2005). In our studies, EDC down-regulated the expression of MMP-9, which might inhibit degradation of mature NGF and lead to the increase of mature NGF. However, the expressions of mature BDNF was also increased, which was not affected by the down-regulation of MMP-9. For this contradictory phenomenon, we cannot find a reasonable explanation now. We find that most of the studies on metabolic pathway of neurotrophins are carried out on neuron cultures instead of astrocytes, which implies that the conversion of pro-neurotrophins to its mature form in astrocytes may be different from neurons (Lee et al., 2001; Gärtner and Staiger, 2002; Hwang et al., 2005). In neurons and neuroendocrine cells, people have found that NGF is mainly trafficked through the constitutive secretory pathway, while BDNF is selectively trafficked through the regulated secretory pathway (Griesbeck et al., 1999; Mowla et al., 1999). However, the process and secretory pathway of NGF and BDNF in astrocytes are rarely reported and the possible differences are worth studying in the future. Besides, as a member of the metzincin family of extracellularly operating proteases, MMP-9 has been found to participates in brain physiology and pathology and contributes to a large variety of brain disorders, including epilepsy, schizophrenia, stroke, neurodegeneration, depression, brain tumors, etc. Therefore, the relationship between antidepressant effect of EDC and MMP-9 regulation will be further studied in the future.

According to the ancient records of traditional Chinese medicine, EDC plays a significant role in “warming kidney yang, tonifying essence, and filling marrow.” These statements are supported by modern research. It has been reported that EDC aqueous extracts can correct the dysfunction of the hypothalamus-pituitary-target axis (adrenal cortical axis, thyroid axis, gonadal axis) in the hydrocortisone-induced kidney yang-deficiency rat model (Jiang et al., 2014). In parallel, EDC aqueous extracts also enhanced learning and memory abilities in the mouse d-galactose-induced aging model and inhibited the activity of monoamine oxidase in brain tissues (Qin et al., 2009). These discoveries suggest that the aqueous extract is an important form of EDC to exert anti-aging, memory promoting, and mood-regulating effects. Previous studies found that EDC aqueous extracts contained a large number of water-soluble peptides exhibiting significant biological activities that could be important active components of EDC (Zhai et al., 2017). Therefore, we plan to study extensively the antidepressant effects of water-soluble proteins and peptides present in EDC to provide a more scientific basis for the utilization of this valuable medicine of animal origin. Besides, compared with abundant antidepressant reports of herbal medicine, there are rare reports on animal medicine. We hope that this study will help to compensate for the lack of research in this area.

## Conclusion

EDC exerted antidepressant effect by improving depression-like behavior in animal models. The effect was linked to the increase in the level of neurotrophic factors in the hippocampus; astrocytes might be the key type of contributing active cells. The aqueous EDC extract was superior to the ethanol extract in promoting the expression of neurotrophic factors. These findings suggest that the aqueous extract of EDC can be used as an adjuvant treatment for patients with depression.

## Data Availability Statement

All datasets presented in this study are included in the article/Supplementary Material.

## Ethics Statement

The animal study was reviewed and approved by the Institutional Animal Care and Use Committee of Nanjing University of Chinese Medicine.

## Author Contributions

YZ and MZ designed the experiments. ML, CC, SQ, XM, CW, and QL performed the experiments including behavioral tests and biochemical analyses. DQ and YD contributed to the preparation of EDC extracts and chemical standardization. ZH and JD contributed to the writing of introduction and discussion. YZ and ML wrote the main manuscript text. YZ and SQ revised the manuscript. All authors have read and approved the submitted manuscript.

## Funding

This research was supported by the National Key R&D Program of China “Study on four development modes of new sources of rare and endangered traditional Chinese Medicine resources” (2018YFC1706100), the National Natural Science Foundation of China (81673720, 81973591), the Open Project Program of Jiangsu Key Laboratory for High Technology Research of TCM Formulae and Jiangsu Collaborative Innovation Center of Chinese Medicinal Resources Industrialization (No. FJGJS-2015–10), and the fifth phase of the 333 high-level personnel training project of Jiangsu Province (BRA2017463) to YZ.

## Conflict of Interest

The authors declare that the research was conducted in the absence of any commercial or financial relationships that could be construed as a potential conflict of interest.
