# Supplementary material for: Elaphuri Davidiani Cornu Improves Depressive-Like Behavior in Mice and Increases Neurotrophic Factor Expression in Mouse Primary Astrocytes via cAMP and ERK-Dependent Pathways
Source: Front Pharmacol. 2020 Nov 16;11:593993. doi: 10.3389/fphar.2020.593993 (PMC7751692; doi:10.3389/fphar.2020.593993)
Supplement: Supplementary file 1 [file datasheet1.docx]

Elaphuri Davidiani Cornu improve depressive-like behaviors on mice and increase neurotrophic factor expressions on mouse primary astrocytes via cAMP and ERK-dependent pathways.

Yue ZHU^1^*^#^, Mengqiu LIU^1#^, Cheng CAO^1^, Suchen QU^1^, Chongqi WEI^1^, Xue-er MENG^1^, Qianyin LOU^1^, Dawei QIAN^1^, Jin-ao DUAN^1^, Yuhua DING^2^, Zhengxiang HAN^3^, Ming ZHAO*

^1^ Jiangsu Key Laboratory for High Technology Research of TCM Formulae and Jiangsu Collaborative Innovation Center of Chinese Medicinal Resources Industrialization, Nanjing University of Chinese Medicine, Nan Jing, Jiangsu Province, China

^2^ Jiangsu Province Dafeng Milu National Nature Reserve, Dafeng, Jiangsu Province, China

^3^ Department of Neurology and Rehabilitation, Shanghai Seventh People's Hospital, Shanghai University of TCM, Shanghai, China

*** Correspondence:**^1^ Dr. Yue ZHU; E-mail: zhuyue@njucm.edu.cn;

^2^ Dr. Ming ZHAO; E-mail: [mingzhao@njucm.edu.cn](mailto:mingzhao@njucm.edu.cn)

^#^ Yue ZHU and Mengqiu LIU contributed equally to this work.

Keywords: Elaphuri Davidiani Cornu; depression; neurotrophic factor; astrocytes; animal medicine

**Supplementary Table 1 Criteria for standardized EDC aqueous extracts**

| **Label** | **Marker chemical** | **EDC aqueous extracts** |
| --- | --- | --- |
| 1 | xanthine | 1.9618 ± 0.0012 ^a^ |
| 2 | hypoxanthine | 1.7263 ± 0.0021 ^a^ |
| 3 | deoxyinosine | 0.6244 ± 0.0009 ^a^ |
| 4 | inosine | 0.8371 ± 0.0026 ^a^ |
| 5 | uracil | 0.5756 ± 0.0031 ^a^ |
| 6 | deoxyuridine | 0.3701 ± 0.0027 ^a^ |
| 7 | uridine | 0.3932 ± 0.0041 ^a^ |
| 8 | thymine | 1.1534 ± 0.0024 ^a^ |
| 9 | thymidine | 0.3619 ± 0.0008 ^a^ |
| 10 | guanine | 0.4123 ± 0.0036 ^a^ |
| 11 | deoxyguanosine | 0.2833 ± 0.0041 ^a^ |
| 12 | guanosine | 0.8793 ± 0.0091 ^a^ |
| 13 | adenine | 0.0114 ± 0.0012 ^a^ |
| 14 | deoxyadenosine | 0.0021 ± 0.0003 ^a^ |
| 15 | adenosine | 0.0157 ± 0.0013 ^a^ |
| 16 | cytidine | 0.2049 ± 0.0053 ^a^ |
| 17 | 2-deoxycytidine | 0.0039 ± 0.0027 ^a^ |

^a^ Values were expressed in μg/g of dried powder of EDC aqueous extracts, Mean ± SEM, n=3.

**Supplementary Table 2. Primers for qPCR analysis**

| **Name** | **Sequence** | **Source** | **Size (bp)** |
| --- | --- | --- | --- |
| NGF-S | CAA TAG CTG CCC GAG TGA CA | NM_013609.3 | 188 |
| NGF-AS | TCC GGT GAG TCC TGT TGA AAG |  |  |
| BDNF-S | TGA CAA CGA CAT CGC ATT AC | NM_007540.4 | 156 |
| BDNF-AS | TTC AGC CGG TCA GAG AAG |  |  |
| MMP-9-S | CTT TGA GGA TCC GCA GAC C | BC046991.1 | 132 |
| MMP-9-S | CTG ACG TGG GTT ACC TCT G |  |  |
| Plasminogen-S | GAC TCA AGG GAC TTT CGG TG | NM_008877.3 | 188 |
| Plasminogen-AS | CTC GAA GCA AAC CAG AGG TC |  |  |
| tPA-S | TGA CAA CGA CAT CGC ATT AC | NM_008872.3 | 186 |
| tPA-AS | TGA CAA CGA CAT CGC ATT AC |  |  |
| neuroserpin-S | TTG GCC CTC ATC AAT GCT GTA | AJ001700.1 | 184 |
| neuroserpin-AS | AGA TAC CAC CAG CCT CAT TGG |  |  |
| TIMP-1-S | CTT TGA GGA TCC GCA GAC C | U54984.1 | 180 |
| TIMP-1-AS | AGC TGT TGC TGA CAA GAT GGT |  |  |
| GAPDH-S | AAC GGA TTT GGC CGT ATT GG | AF106860.2 | 195 |
| GAPDH-AS | CTT CCC GTT CAG CTC TGG G |  |  |
