# Supplementary figures and images for: Elaphuri Davidiani Cornu Improves Depressive-Like Behavior in Mice and Increases Neurotrophic Factor Expression in Mouse Primary Astrocytes via cAMP and ERK-Dependent Pathways
Source: Front Pharmacol. 2020 Nov 16;11:593993. doi: 10.3389/fphar.2020.593993 (PMC7751692; doi:10.3389/fphar.2020.593993)

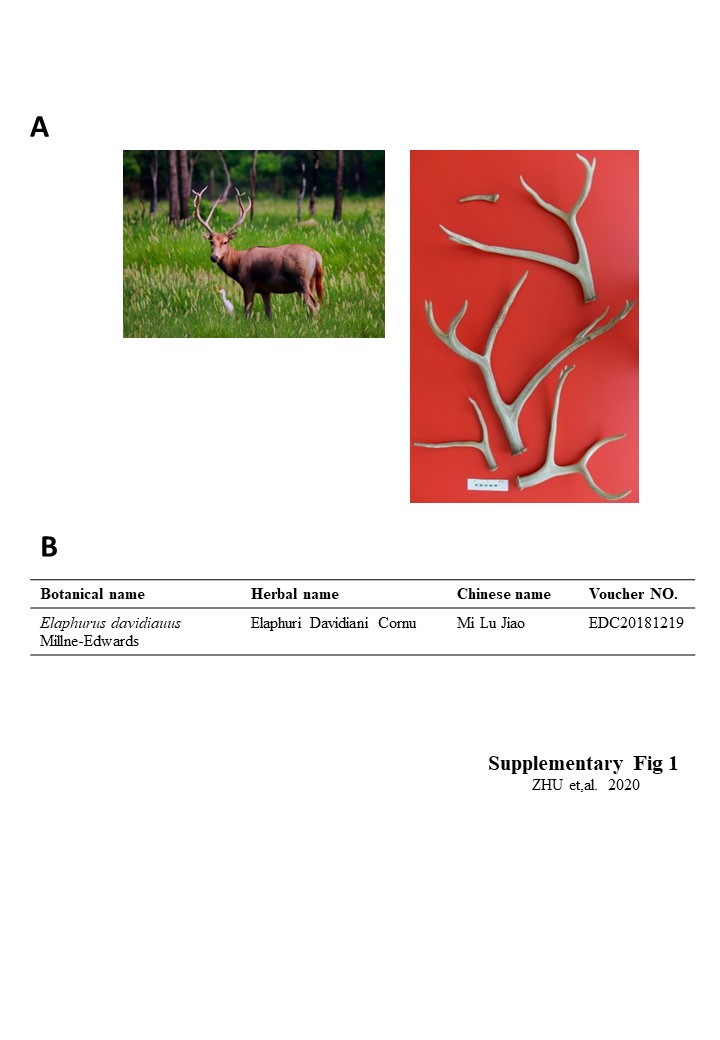

Supplement: Supplementary file 2 [file image1.jpeg]

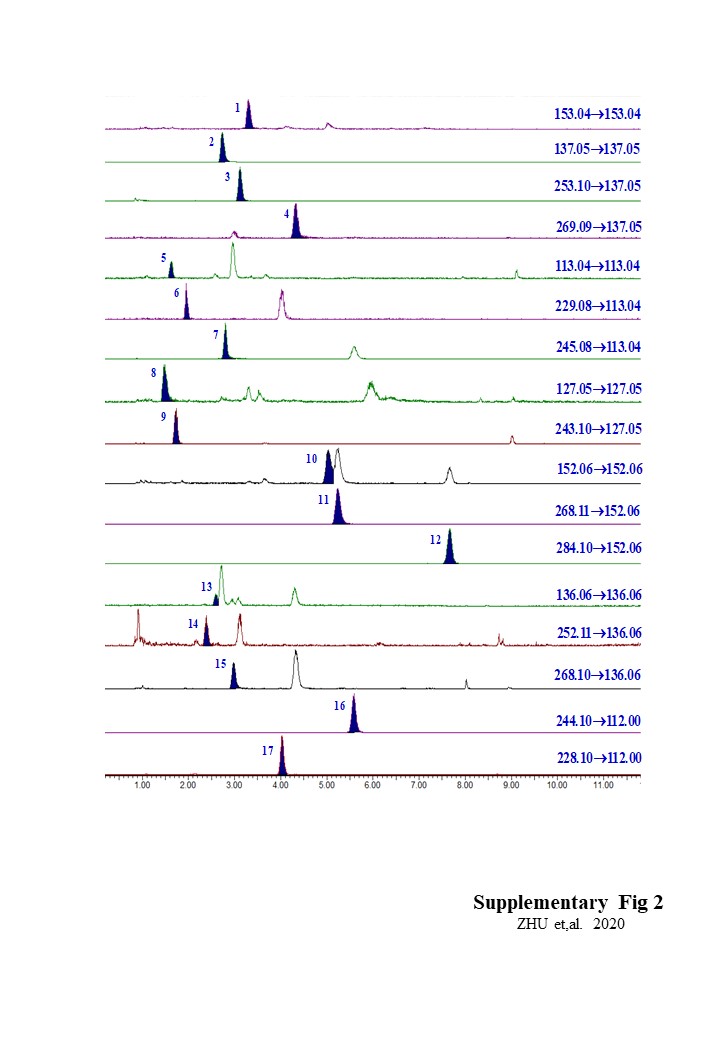

Supplement: Supplementary file 3 [file image2.jpeg]
